# Developing Inpatient Management Strategies for Behavioural and Psychological Symptoms of Dementia (DIMS-BPSD)

**DOI:** 10.1192/bjo.2022.278

**Published:** 2022-06-20

**Authors:** Jennifer Parker, Ryan Beazley, Stephen De Souza

**Affiliations:** Somerset NHS Foundation Trust, Somerset, United Kingdom

## Abstract

**Aims:**

This project details the development of a Quality Improvement Project aiming to review and improve the management of behavioural and psychological symptoms of dementia (BPSD) on an old age psychiatry ward. BPSD refers to a constellation of non-cognitive symptoms and signs which arise in people with dementia, including disturbed perception, thought content, mood or behaviour. Examples include agitation, depression, apathy, repetitive questioning, psychosis, aggression, sleep problems, and socially inappropriate behaviours. BPSD arise in 5/6 of people with dementia over the course of their illness and are associated with a deterioration in cognition and progression in dementia plus secondary harms such as falls and hospitalisation. Pyrland Two ward is a mixed gender specialised organic old age psychiatry inpatient unit serving the county of Somerset. Most patients have a diagnosis of dementia, are being cared for using either MHA or MCA legislation and exhibit one or more BPSD. There was no structured or formalised approach to the management of BPSD at inception.

**Methods:**

1. A point-in-time audit was conducted to produce baseline measurements of BPSD management on the ward, measured against NICE criteria.
2. Plan-Do-Study-Act (PDSA) methodology was employed to incorporate incremental quality improvement interventions such as a ward-round checklist and staff education.

**Results:**

- Baseline: (n = 14) 4/14 formally diagnosed with BPSD. 6/14 were prescribed antipsychotic medications, of which 1/6 fully met NICE standards. 2/14 had structured assessment tools used.
- Results following introduction of improvement methods: (n = 8) 8/8 formally diagnosed with BPSD. 7/8 were prescribed antipsychotic medications, of which 4/7 fully met NICE standards. 7/8 had structured assessment tools used.

**Conclusion:**

It was possible to see modest improvements in the ward-based management of BPSD using quality improvement methodology, including more favourable psychotropic prescribing. However, total patient numbers are small and further interventions, such as more PDSA cycles, may add value and encourage sustainability.

